# *N*′-(2-Furylmethyl­ene)nicotinohydrazide

**DOI:** 10.1107/S1600536809033698

**Published:** 2009-08-29

**Authors:** Heng-yu Qian, Zhi-gang Yin, Chun-xia Zhang, Zhi-qiang Yao

**Affiliations:** a Key Laboratory of Surface and Interface Science of Henan, School of Materials & Chemical Engineering, Zhengzhou University of Light Industry, Zhengzhou 450002, People’s Republic of China

## Abstract

The asymmetric unit of the title compound, C_11_H_9_N_3_O_2_, contains two independent mol­ecules: the dihedral angles between the pyridine ring and the furyl ring are 17.00 (16) and 34.12 (15)°. The crystal structure involves inter­molecular C—H⋯O, N—H⋯N and N—H⋯O hydrogen bonds.

## Related literature

For the role played by Schiff bases in the development of various proteins and enzymes, see: Kahwa *et al.* (1986 ▶); Santos *et al.* (2001 ▶).
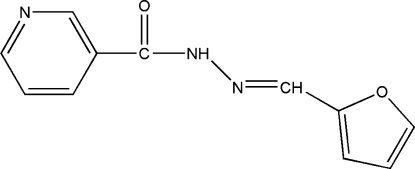


## Experimental

### 

#### Crystal data

C_11_H_9_N_3_O_2_
*M* *_r_* = 215.21
Monoclinic, 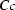

*a* = 17.4363 (3) Å
*b* = 16.9143 (3) Å
*c* = 7.9639 (1) Å
β = 115.326 (1)°
*V* = 2122.99 (6) Å^3^
*Z* = 8
Mo *K*α radiation
μ = 0.10 mm^−1^
*T* = 296 K
0.34 × 0.24 × 0.15 mm

#### Data collection

Bruker SMART CCD area-detector diffractometer
Absorption correction: multi-scan (*SADABS*; Bruker, 1998 ▶) *T*_min_ = 0.964, *T*_max_ = 0.981
9336 measured reflections
2443 independent reflections
1908 reflections with *I* > 2σ(*I*)
*R*_int_ = 0.026

#### Refinement

*R*[*F* ^2^ > 2σ(*F* ^2^)] = 0.035
*wR*(*F* ^2^) = 0.086
*S* = 1.06
2443 reflections
290 parameters
2 restraints
H-atom parameters constrained
Δρ_max_ = 0.10 e Å^−3^
Δρ_min_ = −0.16 e Å^−3^

### 

Data collection: *SMART* (Bruker, 1998 ▶); cell refinement: *SAINT* (Bruker, 1998 ▶); data reduction: *SAINT*; program(s) used to solve structure: *SHELXS97* (Sheldrick, 2008 ▶); program(s) used to refine structure: *SHELXL97* (Sheldrick, 2008 ▶); molecular graphics: *SHELXTL* (Sheldrick, 2008 ▶); software used to prepare material for publication: *SHELXTL*.

## Supplementary Material

Crystal structure: contains datablocks global, I. DOI: 10.1107/S1600536809033698/hg2558sup1.cif

Structure factors: contains datablocks I. DOI: 10.1107/S1600536809033698/hg2558Isup2.hkl

Additional supplementary materials:  crystallographic information; 3D view; checkCIF report

## Figures and Tables

**Table 1:** Hydrogen-bond geometry (Å, °)

*D*—H⋯*A*	*D*—H	H⋯*A*	*D*⋯*A*	*D*—H⋯*A*
N2—H2*A*⋯O4^i^	0.86	2.26	3.080 (2)	161
N2—H2*A*⋯N4^i^	0.86	2.51	3.112 (3)	128
N5—H5*A*⋯O2^ii^	0.86	2.01	2.843 (3)	162
C8—H8*A*⋯O4^i^	0.93	2.56	3.433 (3)	156
C16—H16*A*⋯O2^ii^	0.93	2.45	3.228 (3)	141
C22—H22*A*⋯O2^ii^	0.93	2.43	3.260 (3)	149

Symmetry codes: (i) 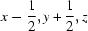; (ii) 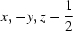.

**Fig. 1.**
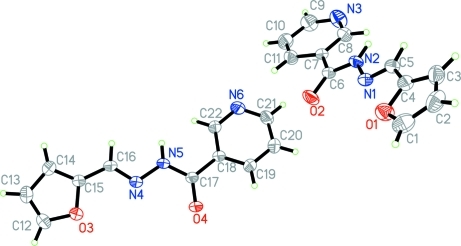
the ORTEP plot of (I). Displacement ellipsoids are drawn at the 30% probability level.

## supplementary crystallographic information

### Comment

The chemistry of Schiff bases has attracted a great deal of interest in recent years. These compounds play an important role in the development of various proteins and enzymes (Kahwa *et al.*, 1986; Santos *et al.*, 2001). As part of our in the study of the coordination chemistry of Schiff bases, we synthesized the title compound and determined its crystal structure.

The molecular structure is shown in Fig.1. Each molecule is not planar, making the dihedral angle of 17.00 (16) and 34.12 (15)° between pyridine and furyl rings, respectively. In the crystal structure, molecules are linked through intermolecular C—H···O, N—H···N and N—H···O hydrogen bonds, forming a network.

### Experimental

Pyridine-4-carboxylic acid hydrazide (1 mmol, 0.137 g) was dissolved in anhydrous ethanol (15 ml), The mixture was stirred for several minutes at 351 K. Furan-2-carbaldehyde (1 mmol, 0.96 g) in ethanol (8 mm l) was added dropwise and the mixture was stirred at refluxing temperature for 2 h. The product was isolated and recrystallized from methanol. Pink single crystals of (I) was obtained after 3 d.

### Refinement

All H atoms were positioned geometrically and refined as riding with C—H = 0.93(aromatic) and N—H = 0.86Å, with *U*_iso_(H) = 1.2*U*_eq_(CH, NH).

### Crystal data

**Table:**

C_11_H_9_N_3_O_2_	*F*(000) = 896
*M**_r_* = 215.21	*D*_x_ = 1.347 Mg m^−^^3^
Monoclinic, *C**c*	Mo *K*α radiation, λ = 0.71073 Å
Hall symbol: C -2yc	Cell parameters from 2878 reflections
*a* = 17.4363 (3) Å	θ = 2.4–26.0°
*b* = 16.9143 (3) Å	µ = 0.10 mm^−^^1^
*c* = 7.9639 (1) Å	*T* = 296 K
β = 115.326 (1)°	Block, pink
*V* = 2122.99 (6) Å^3^	0.34 × 0.24 × 0.15 mm
*Z* = 8	

### Data collection

**Table:**

Bruker SMART CCD area-detector diffractometer	2443 independent reflections
Radiation source: fine-focus sealed tube	1908 reflections with *I* > 2σ(*I*)
graphite	*R*_int_ = 0.026
ω scans	θ_max_ = 27.5°, θ_min_ = 1.8°
Absorption correction: multi-scan (*SADABS*; Bruker, 1998)	*h* = −22→15
*T*_min_ = 0.964, *T*_max_ = 0.981	*k* = −20→21
9336 measured reflections	*l* = −7→10

### Refinement

**Table:**

Refinement on *F*^2^	Primary atom site location: structure-invariant direct methods
Least-squares matrix: full	Secondary atom site location: difference Fourier map
*R*[*F*^2^ > 2σ(*F*^2^)] = 0.035	Hydrogen site location: inferred from neighbouring sites
*wR*(*F*^2^) = 0.086	H-atom parameters constrained
*S* = 1.06	*w* = 1/[σ^2^(*F*_o_^2^) + (0.0488*P*)^2^] where *P* = (*F*_o_^2^ + 2*F*_c_^2^)/3
2443 reflections	(Δ/σ)_max_ = 0.006
290 parameters	Δρ_max_ = 0.10 e Å^−^^3^
2 restraints	Δρ_min_ = −0.16 e Å^−^^3^

### Special details

**Table:**

Geometry. All e.s.d.'s (except the e.s.d. in the dihedral angle between two l.s. planes) are estimated using the full covariance matrix. The cell e.s.d.'s are taken into account individually in the estimation of e.s.d.'s in distances, angles and torsion angles; correlations between e.s.d.'s in cell parameters are only used when they are defined by crystal symmetry. An approximate (isotropic) treatment of cell e.s.d.'s is used for estimating e.s.d.'s involving l.s. planes.
Refinement. Refinement of *F*^2^ against ALL reflections. The weighted *R*-factor *wR* and goodness of fit *S* are based on *F*^2^, conventional *R*-factors *R* are based on *F*, with *F* set to zero for negative *F*^2^. The threshold expression of *F*^2^ > σ(*F*^2^) is used only for calculating *R*-factors(gt) *etc*. and is not relevant to the choice of reflections for refinement. *R*-factors based on *F*^2^ are statistically about twice as large as those based on *F*, and *R*- factors based on ALL data will be even larger.

### Fractional atomic coordinates and isotropic or equivalent isotropic displacement parameters (Å^2^)

**Table:**

	*x*	*y*	*z*	*U*_iso_*/*U*_eq_	
N1	0.20002 (12)	0.30973 (11)	0.8151 (3)	0.0512 (4)	
N2	0.15397 (11)	0.29763 (11)	0.6278 (2)	0.0500 (4)	
H2A	0.1101	0.3262	0.5653	0.060*	
O4	0.52299 (10)	−0.06828 (10)	0.4681 (3)	0.0659 (5)	
N4	0.45709 (11)	−0.21482 (11)	0.4338 (2)	0.0505 (5)	
N5	0.40911 (11)	−0.14890 (11)	0.3504 (3)	0.0522 (5)	
H5A	0.3552	−0.1532	0.2855	0.063*	
C6	0.17831 (13)	0.24079 (13)	0.5439 (3)	0.0503 (5)	
C17	0.44693 (14)	−0.07807 (13)	0.3711 (3)	0.0490 (5)	
O1	0.27842 (12)	0.33154 (12)	1.1928 (2)	0.0715 (5)	
O2	0.23970 (12)	0.19833 (11)	0.6284 (3)	0.0763 (6)	
C7	0.12722 (13)	0.23106 (12)	0.3412 (3)	0.0461 (5)	
O3	0.53896 (10)	−0.35845 (9)	0.5715 (2)	0.0622 (4)	
C18	0.39031 (13)	−0.01116 (13)	0.2696 (3)	0.0478 (5)	
C15	0.45280 (14)	−0.35168 (14)	0.4952 (3)	0.0532 (5)	
C5	0.17080 (15)	0.36173 (14)	0.8885 (3)	0.0539 (6)	
H5B	0.1217	0.3892	0.8143	0.065*	
C16	0.41431 (15)	−0.27691 (14)	0.4225 (3)	0.0553 (6)	
H16A	0.3554	−0.2738	0.3644	0.066*	
C4	0.21241 (16)	0.37849 (14)	1.0833 (4)	0.0572 (6)	
C8	0.07516 (16)	0.28882 (14)	0.2286 (3)	0.0589 (6)	
H8A	0.0719	0.3366	0.2829	0.071*	
N3	0.02917 (18)	0.28065 (15)	0.0467 (3)	0.0813 (7)	
C21	0.28729 (18)	0.10962 (16)	0.0818 (4)	0.0692 (7)	
H21A	0.2520	0.1515	0.0195	0.083*	
C11	0.13288 (17)	0.16115 (14)	0.2577 (4)	0.0650 (7)	
H11A	0.1678	0.1206	0.3283	0.078*	
N6	0.26106 (14)	0.03697 (14)	0.0239 (3)	0.0773 (7)	
C14	0.41892 (19)	−0.42120 (16)	0.5088 (5)	0.0781 (8)	
H14A	0.3614	−0.4324	0.4666	0.094*	
C19	0.41546 (17)	0.06558 (14)	0.3224 (4)	0.0628 (6)	
H19A	0.4676	0.0759	0.4214	0.075*	
C22	0.31279 (16)	−0.02186 (15)	0.1181 (4)	0.0651 (7)	
H22A	0.2956	−0.0734	0.0793	0.078*	
C3	0.1971 (2)	0.43309 (15)	1.1883 (5)	0.0737 (8)	
H3B	0.1554	0.4720	1.1465	0.088*	
C9	0.0358 (2)	0.2127 (2)	−0.0274 (4)	0.0796 (8)	
H9A	0.0036	0.2059	−0.1543	0.095*	
C20	0.36299 (19)	0.12676 (15)	0.2276 (4)	0.0705 (7)	
H20A	0.3788	0.1789	0.2621	0.085*	
C13	0.4863 (2)	−0.47379 (17)	0.5986 (5)	0.0821 (9)	
H13A	0.4821	−0.5263	0.6282	0.098*	
C12	0.5564 (2)	−0.43447 (15)	0.6328 (4)	0.0706 (7)	
H12A	0.6107	−0.4557	0.6911	0.085*	
C1	0.3036 (2)	0.3593 (2)	1.3692 (4)	0.0870 (9)	
H1B	0.3482	0.3383	1.4732	0.104*	
C10	0.0865 (2)	0.15199 (17)	0.0696 (4)	0.0729 (8)	
H10A	0.0897	0.1056	0.0102	0.087*	
C2	0.2564 (2)	0.4201 (2)	1.3734 (5)	0.0848 (9)	
H2B	0.2614	0.4485	1.4776	0.102*	

### Atomic displacement parameters (Å^2^)

**Table:**

	*U*^11^	*U*^22^	*U*^33^	*U*^12^	*U*^13^	*U*^23^
N1	0.0387 (9)	0.0551 (10)	0.0519 (11)	0.0008 (8)	0.0119 (9)	0.0000 (8)
N2	0.0347 (9)	0.0544 (10)	0.0500 (10)	0.0077 (8)	0.0078 (8)	0.0012 (8)
O4	0.0354 (8)	0.0574 (9)	0.0832 (12)	−0.0024 (7)	0.0045 (8)	−0.0060 (8)
N4	0.0369 (9)	0.0520 (11)	0.0563 (11)	0.0020 (8)	0.0137 (9)	0.0018 (8)
N5	0.0318 (9)	0.0514 (11)	0.0601 (12)	0.0021 (8)	0.0070 (8)	0.0029 (8)
C6	0.0331 (11)	0.0486 (12)	0.0574 (13)	0.0030 (9)	0.0080 (10)	−0.0008 (10)
C17	0.0360 (11)	0.0504 (12)	0.0527 (12)	−0.0015 (9)	0.0115 (10)	−0.0065 (9)
O1	0.0645 (11)	0.0809 (12)	0.0594 (10)	0.0077 (10)	0.0171 (9)	−0.0046 (9)
O2	0.0533 (10)	0.0745 (11)	0.0689 (11)	0.0264 (9)	−0.0046 (9)	−0.0126 (9)
C7	0.0331 (10)	0.0472 (11)	0.0530 (12)	−0.0021 (9)	0.0138 (10)	0.0000 (9)
O3	0.0452 (9)	0.0555 (9)	0.0781 (11)	0.0037 (8)	0.0189 (9)	0.0055 (8)
C18	0.0370 (11)	0.0504 (12)	0.0522 (12)	−0.0014 (9)	0.0154 (10)	−0.0022 (9)
C15	0.0411 (12)	0.0536 (13)	0.0572 (13)	−0.0022 (10)	0.0138 (11)	0.0001 (10)
C5	0.0457 (12)	0.0527 (12)	0.0616 (15)	0.0026 (10)	0.0214 (12)	0.0041 (10)
C16	0.0355 (11)	0.0578 (14)	0.0616 (14)	−0.0020 (10)	0.0104 (10)	0.0026 (11)
C4	0.0527 (14)	0.0565 (13)	0.0638 (14)	−0.0025 (11)	0.0264 (12)	0.0006 (11)
C8	0.0576 (15)	0.0628 (14)	0.0552 (14)	0.0096 (12)	0.0232 (12)	0.0058 (11)
N3	0.0926 (19)	0.0879 (16)	0.0512 (12)	0.0251 (14)	0.0192 (12)	0.0123 (12)
C21	0.0615 (17)	0.0575 (15)	0.0865 (19)	0.0119 (13)	0.0298 (15)	0.0192 (13)
C11	0.0587 (15)	0.0573 (13)	0.0621 (15)	0.0076 (12)	0.0097 (12)	−0.0028 (11)
N6	0.0512 (13)	0.0676 (14)	0.0881 (16)	0.0060 (11)	0.0060 (12)	0.0147 (12)
C14	0.0587 (16)	0.0559 (15)	0.113 (2)	−0.0062 (13)	0.0302 (17)	0.0049 (14)
C19	0.0551 (14)	0.0523 (14)	0.0685 (15)	−0.0050 (11)	0.0145 (12)	−0.0060 (11)
C22	0.0495 (14)	0.0530 (13)	0.0729 (16)	−0.0022 (11)	0.0073 (13)	0.0017 (12)
C3	0.081 (2)	0.0624 (15)	0.086 (2)	−0.0060 (14)	0.0431 (18)	−0.0135 (14)
C9	0.080 (2)	0.094 (2)	0.0501 (14)	0.0059 (17)	0.0138 (15)	0.0000 (14)
C20	0.0716 (18)	0.0484 (13)	0.0849 (19)	−0.0006 (13)	0.0272 (16)	0.0019 (12)
C13	0.082 (2)	0.0527 (15)	0.110 (3)	0.0018 (15)	0.0400 (19)	0.0111 (15)
C12	0.0660 (17)	0.0595 (15)	0.0796 (17)	0.0141 (13)	0.0246 (14)	0.0079 (13)
C1	0.078 (2)	0.107 (3)	0.0601 (18)	−0.015 (2)	0.0146 (16)	−0.0101 (16)
C10	0.0745 (18)	0.0722 (18)	0.0628 (16)	−0.0057 (15)	0.0206 (14)	−0.0127 (13)
C2	0.103 (2)	0.082 (2)	0.082 (2)	−0.0289 (18)	0.052 (2)	−0.0266 (16)

### Geometric parameters (Å, °)

**Table:**

N1—C5	1.277 (3)	C8—N3	1.329 (3)
N1—N2	1.374 (3)	C8—H8A	0.9300
N2—C6	1.339 (3)	N3—C9	1.319 (4)
N2—H2A	0.8600	C21—N6	1.323 (4)
O4—C17	1.227 (3)	C21—C20	1.365 (4)
N4—C16	1.269 (3)	C21—H21A	0.9300
N4—N5	1.382 (2)	C11—C10	1.374 (4)
N5—C17	1.343 (3)	C11—H11A	0.9300
N5—H5A	0.8600	N6—C22	1.337 (3)
C6—O2	1.224 (3)	C14—C13	1.401 (4)
C6—C7	1.482 (3)	C14—H14A	0.9300
C17—C18	1.492 (3)	C19—C20	1.374 (4)
O1—C4	1.363 (3)	C19—H19A	0.9300
O1—C1	1.363 (4)	C22—H22A	0.9300
C7—C8	1.374 (3)	C3—C2	1.409 (5)
C7—C11	1.380 (3)	C3—H3B	0.9300
O3—C15	1.363 (3)	C9—C10	1.360 (4)
O3—C12	1.363 (3)	C9—H9A	0.9300
C18—C19	1.377 (3)	C20—H20A	0.9300
C18—C22	1.386 (3)	C13—C12	1.315 (4)
C15—C14	1.341 (4)	C13—H13A	0.9300
C15—C16	1.433 (3)	C12—H12A	0.9300
C5—C4	1.432 (4)	C1—C2	1.327 (5)
C5—H5B	0.9300	C1—H1B	0.9300
C16—H16A	0.9300	C10—H10A	0.9300
C4—C3	1.346 (4)	C2—H2B	0.9300
			
C5—N1—N2	115.95 (18)	N6—C21—H21A	118.0
C6—N2—N1	119.08 (17)	C20—C21—H21A	118.0
C6—N2—H2A	120.5	C10—C11—C7	119.5 (2)
N1—N2—H2A	120.5	C10—C11—H11A	120.3
C16—N4—N5	114.65 (17)	C7—C11—H11A	120.3
C17—N5—N4	119.75 (17)	C21—N6—C22	116.5 (2)
C17—N5—H5A	120.1	C15—C14—C13	107.2 (3)
N4—N5—H5A	120.1	C15—C14—H14A	126.4
O2—C6—N2	122.4 (2)	C13—C14—H14A	126.4
O2—C6—C7	120.7 (2)	C20—C19—C18	119.5 (2)
N2—C6—C7	116.88 (18)	C20—C19—H19A	120.3
O4—C17—N5	122.6 (2)	C18—C19—H19A	120.3
O4—C17—C18	121.7 (2)	N6—C22—C18	124.3 (2)
N5—C17—C18	115.71 (18)	N6—C22—H22A	117.8
C4—O1—C1	105.7 (2)	C18—C22—H22A	117.8
C8—C7—C11	117.3 (2)	C4—C3—C2	106.9 (3)
C8—C7—C6	123.6 (2)	C4—C3—H3B	126.5
C11—C7—C6	119.08 (19)	C2—C3—H3B	126.5
C15—O3—C12	105.9 (2)	N3—C9—C10	124.3 (3)
C19—C18—C22	117.0 (2)	N3—C9—H9A	117.8
C19—C18—C17	119.9 (2)	C10—C9—H9A	117.8
C22—C18—C17	123.1 (2)	C21—C20—C19	118.8 (2)
C14—C15—O3	109.3 (2)	C21—C20—H20A	120.6
C14—C15—C16	131.3 (2)	C19—C20—H20A	120.6
O3—C15—C16	119.4 (2)	C12—C13—C14	106.7 (2)
N1—C5—C4	121.1 (2)	C12—C13—H13A	126.7
N1—C5—H5B	119.5	C14—C13—H13A	126.7
C4—C5—H5B	119.5	C13—C12—O3	110.9 (3)
N4—C16—C15	122.9 (2)	C13—C12—H12A	124.5
N4—C16—H16A	118.6	O3—C12—H12A	124.5
C15—C16—H16A	118.6	C2—C1—O1	111.3 (3)
C3—C4—O1	109.9 (2)	C2—C1—H1B	124.4
C3—C4—C5	131.7 (3)	O1—C1—H1B	124.4
O1—C4—C5	118.4 (2)	C9—C10—C11	118.1 (3)
N3—C8—C7	124.0 (2)	C9—C10—H10A	121.0
N3—C8—H8A	118.0	C11—C10—H10A	121.0
C7—C8—H8A	118.0	C1—C2—C3	106.2 (3)
C9—N3—C8	116.8 (2)	C1—C2—H2B	126.9
N6—C21—C20	123.9 (2)	C3—C2—H2B	126.9

### Hydrogen-bond geometry (Å, °)

**Table:**

*D*—H···*A*	*D*—H	H···*A*	*D*···*A*	*D*—H···*A*
N2—H2A···O4^i^	0.86	2.26	3.080 (2)	161
N2—H2A···N4^i^	0.86	2.51	3.112 (3)	128
N5—H5A···O2^ii^	0.86	2.01	2.843 (3)	162
C8—H8A···O4^i^	0.93	2.56	3.433 (3)	156
C16—H16A···O2^ii^	0.93	2.45	3.228 (3)	141
C22—H22A···O2^ii^	0.93	2.43	3.260 (3)	149

Symmetry codes: (i) *x*−1/2, *y*+1/2, *z*; (ii) *x*, −*y*, *z*−1/2.
